# Abnormal karyotype is an independent predictor of inferior survival in Blastic Plasmacytoid Dendritic Cell Neoplasm (BPDCN)

**DOI:** 10.1038/s41408-023-00812-y

**Published:** 2023-03-13

**Authors:** Mostafa Abdallah, Kristen McCullough, Rimal Ilyas, Kebede H. Begna, Aref Al-Kali, Mark R. Litzow, William J. Hogan, Abhishek Mangaonkar, Hassan Alkhateeb, Mithun V. Shah, Michelle A. Elliott, James M. Foran, Talha Badar, Jeanne M. Palmer, Cecilia Arana Yi, Lisa Sproat, Animesh Pardanani, Mrinal M. Patnaik, Horatiu Olteanu, Rhett P. Ketterling, Ayalew Tefferi, Naseema Gangat

**Affiliations:** 1 Division of Hematology, Mayo Clinic, Rochester, MN USA; 2 Division of Hematology, Mayo Clinic, Jacksonville, FL USA; 3 Division of Hematology, Mayo Clinic, Scottsdale, AZ USA; 4 Division of Hematopathology, Mayo Clinic, Rochester, MN USA; 5 Division of Laboratory Medicine and Cytogenetics, Mayo Clinic, Rochester, MN USA

**Keywords:** Acute myeloid leukaemia, Leukaemia

Dear Editor,

Blastic plasmacytoid dendritic cell neoplasm (BPDCN), formerly known as agranular CD4^+^/CD56^+^ hematodermic neoplasm or blastic natural killer (NK) cell lymphoma was officially recognized as a distinct entity in the 2016 revision of the World Health Organization (WHO) classification of myeloid malignancies [1]. It predominantly afflicts older males (median age; 66–70 years), displaying cutaneous, lymph node, bone marrow and/or central nervous system (CNS) involvement [2]. Historically, patients with BPDCN were treated with chemotherapy regimens used for acute lymphoblastic leukemia (ALL), acute myeloid leukemia (AML), and lymphoma. However, in recent years, treatment paradigms have evolved with FDA-approval of tagraxofusp, a CD123 directed antibody conjugate composed of human interleukin-3 (IL-3) and truncated diphtheria toxin for BPDCN and venetoclax (bcl-2 inhibitor) for AML [3, 4]. In an open-label clinical trial with tagraxofusp in 47 patients with BPDCN, response rates were 90 and 67% among treatment naïve and relapsed patients, respectively [5]. Nonetheless, despite high response rates, tagraxofusp has not been shown to confer a survival benefit and prognosis for patients with BPDCN continues to be uniformly poor. A retrospective study of one hundred patients with BPDCN, disclosed similar survival among patients that received frontline hyper CVAD (hyper fractioned cyclophosphamide, vincristine, doxorubicin, dexamethasone, alternating with high-dose methotrexate and cytarabine), tagraxofusp or other chemotherapy regimens [6]. On the other hand, allogeneic stem cell transplantation (ASCT) has been consistently shown to prolong survival in patients with BPDCN with 3-year survival rates as high as 72.4% [7–10]. Additional predictors of survival noted in some studies include advanced age [2, 8], presence of disseminated disease [8], extranodal involvement [8], and abnormal karyotype [2]. Accordingly, the current study, was conducted with the following objectives i) describe characteristics and treatment patterns for BPDCN patients evaluated at the Mayo Clinic over the last two decades, and ii) determine survival outcomes including predictors of survival in the context of treatments received.

In the current study, 58 consecutive patients with BPDCN evaluated at the Mayo Clinic (Rochester MN, Arizona, Florida) between April 2000 and April 2022 were retrospectively recruited following institutional review board approval. Follow-up information was updated in January 2023. Diagnosis of BPDCN was retrospectively established through review of skin, lymph node or bone marrow biopsy [1]. In addition, majority of patients underwent computed tomography (CT) or positron emission tomography (PET) imaging and cerebrospinal fluid sampling to evaluate for nodal and CNS disease [11]. Cytogenetic studies were performed on bone marrow samples in a subset of patients by conventional karyotype, and reported according to the 2021 International System for Human Cytogenetic Nomenclature [12]. Response assessment was performed per treating physician discretion with complete response defined as the disappearance of the disease in each involved site either by physical exam, imaging studies and/or biopsies. Bone marrow response was assessed according to the 2017 European Leukemia Net (ELN) criteria [13]. Overall survival was evaluated by the Kaplan–Meier method with differences compared by the log-rank test and predictors of survival determined by Cox proportional hazards model. All analyses were performed using JMP Pro 16.0.0 software package, SAS Institute, Cary, NC.

A total of 58 patients with BPDCN (median age 69 years, range 19–76; 79% males), presented with cutaneous (*n* = 37, 64%), bone marrow (*n* = 37, 64%), lymph node (*n* = 27, 47%) and CNS disease (*n* = 11, 19%). 8 of 36 (22%) evaluable patients had abnormal karyotype other than loss of Y chromosome (-Y), ELN cytogenetic risk distribution was intermediate and adverse in four patients each; among the latter, all were classified as complex and monosomal. Supplemental Table 1 provides details regarding each specific cytogenetic abnormality. Follow-up cytogenetic studies were performed in a subset of patients (*n* = 21), of which 5 (24%) patients demonstrated clonal evolution at the time of relapse (Supplemental Table 2). Mutational analysis was performed in a subset of patients (*n* = 9) and revealed mutations in *ASXL1* (*n* = 6), *RUNX1* (*n* = 6), *TET2* (*n* = 5), *SRSF2* (*n* = 3), *NRAS* (*n* = 2), and *ZRSR2* (*n* = 1). Table 1 summarizes clinical characteristics including treatment details for all fifty-eight BPDCN patients of which 41 (71%) received one or more cycles of chemotherapy. Frontline treatments included hyper CVAD (*n* = 12, 21%) AML induction (*n* = 8, 14%), lymphoma regimen (*n* = 7, 12%), tagraxofusp (*n* = 5, 9%), hypomethylating agent with (*n* = 4, 7%) or without venetoclax (*n* = 3, 5%), fludarabine (*n* = 1) and bortezomib (*n* = 1). Complete response was achieved in 24 of 41(59%) treated patients which included complete remission (CR) (*n* = 17) and CR with incomplete count recovery (CRi) (*n* = 7) while an additional 2 patients achieved partial remission (PR). Relapse after a median response duration of 8 months was documented in 19 of 24 (79%) responding patients. Second-line therapies are detailed in Table 1, commonly utilized regimens included AML induction (*n* = 7), tagraxofusp (*n* = 3), lymphoma regimen (*n* = 3), hyper CVAD (*n* = 2) and hypomethylating agents plus venetoclax (*n* = 2). Three patients underwent autologous stem cell transplantation while in CRi. On the other hand, a total of 15 (26%) patients (median age; 62 years, range-20–72 years) underwent ASCT in first remission (*n* = 12) or following relapse (*n* = 3), all patients were in CR (*n* = 9) or CRi (*n* = 6) at the time of transplant. Donor sources included matched unrelated (*n* = 10), match related (*n* = 3), haplo-identical (*n* = 1) and mismatched unrelated (*n* = 1), with reduced intensity conditioning utilized in 10 patients. Among patients that were transplanted, five and six patients had received tagraxofusp and hyper CVAD, respectively. Correspondingly, three and seven of non-transplanted patients had exposure to tagraxofusp and hyper CVAD.

**Table 1:** Presenting clinical and laboratory characteristics and treatment details of 58 adult patients with blastic plasmacytoid dendritic cell neoplasm.

Variables	All patients *n* = 58
Age in years, median (range),	69 (58–76)
Age >70 years, *n* (%)	25 (43)
Male, *n* (%)	46 (79)
Hemoglobin, g/dl, median (range)	11.6 (9.5–13.5)
Leukocyte count × 10^9^/L, median (range)	4.4 (2.1–6.9)
Platelet count × 10^9^/L, median (range)	132 (67–204)
Bone marrow blast percentage, median (range)	50 (1–95%)
Site of involvement, *n* (%)	
Cutaneous	37 (64)
Bone marrow	37 (64)
Lymph node	27 (47)
Central nervous system	11(19)
Abnormal karyotype, *n* (%)	8/36 (22)
ELN 2017 risk	
Intermediate	4/36 (11)
Adverse	4/36 (11)
Mutations on NGS, *n* (%)	*n* = 9
* ASXL1*	6 (67)
* RUNX1*	6 (67)
* TET2*	5 (56)
* SRSF2*	3 (33)
* NRAS*	2 (22)
* ZRSR2*	1(11)
First-line treatment, *n* (%)	41 (71)
Hyper CVAD	12 (21)
AML induction regimen^a^	8(14)
Lymphoma regimen^b^	7 (12)
Tagraxofusp	5(9)
Hypomethylating agent + venetoclax	4(7)
Hypomethylating agent	3(5)
Other chemotherapy^c^	2 (3)
Initial treatment response, *n* (%)	
Complete remission (CR) with or without count recovery (CRi)	24/41 (59)
Partial remission (PR)	2/41 (5)
Relapse after first remission	19/24 (79)
Second-line treatment, *n* (%)	19(33)
AML induction regimen^a^	7(12)
Tagraxofusp	3 (5)
Lymphoma regimen^b^	3(5)
Hypomethylating agent + venetoclax	2 (5)
Hyper CVAD	2(3)
Hypomethylating agent	1 (2)
Other chemotherapy^c^	1(2)
Autologous stem cell transplant, *n* (%)	3(5)
Allogeneic stem cell transplant, *n* (%)	15(26)

*ELN* European LeukemiaNet, *NGS* next generation sequencing, *AML* acute myeloid leukemia, *Hyper CVAD* hyper fractioned cyclophosphamide, vincristine, doxorubicin, dexamethasone alternating with high dose methotrexate and cytarabine.

^a^AML induction regimen- 7 (cytarabine)+3 (daunorubicin/idarubicin), CLAM (cladribine, cytarabine, mitoxantrone), FLAM (fludarabine. cytarabine, mitoxantrone), mitoxantrone and cytarabine, ADE (cytarabine, daunorubicin, etoposide), high dose cytarabine monotherapy.

^b^Lymphoma regimen- CHOP (cyclophosphamide, doxorubicin, vincristine, prednisone), ICE (cyclophosphamide, cisplatin, etoposide), cyclophosphamide, etoposide, and bleomycin.

^c^Other chemotherapy- single agent fludarabine, bortezomib, or lenalidomide.

After a median follow up of 13 months (range, 1–115 months), 47 (81%) patients have died. Among 15 patients that underwent ASCT, 8 have died from disease relapse (*n* = 5) or graft versus host disease (*n* = 3). On the other hand, among the non-transplanted patients with complete follow-up (*n* = 40), all have died. Overall median survival was 14 months (95% CI; 6–28 months) with 1/3/5-year survival rates of 57%/16%/5% and was superior in patients that underwent ASCT vs those not transplanted (27 vs 11 months; *p* = 0.002, 1 /3/5-year survival, 93%/33%/13%, vs 48%/12%/0%) (Fig. 1a). On univariate analysis, overall survival was inferior in patients older than 70 years of age (11 vs 17 months, *p* = 0.03), male patients (13 vs 35 months; *p* = 0.01), presence of abnormal karyotype (11 vs 23 months; *p* = 0.02), and absence of CR/CRi (11 vs 19 months; *p* = 0.03). On the other hand, survival was not significantly different in patients with exposure to tagraxofusp (20 vs 13 months, *p* = 0.63) or hyper CVAD (23 vs 13 months, *p* = 0.21). Multivariable analysis confirmed the prognostic impact of abnormal karyotype (*p* = 0.01, HR, 3.7, 95% CI (1.4–9.7) and ASCT (*p* = 0.02, HR 0.4, 95% CI (0.1–0.8) on overall survival (Fig. 1a, b). Moreover, survival of patients with abnormal karyotype was inferior to non-transplanted patients with normal karyotype (median survival, 11 months, 1/3-year survival 38%/ 0% and 14 months, 1/3/5-year survival 67%/22%/0%, respectively, *p* = 0.08).

**Fig. 1:**
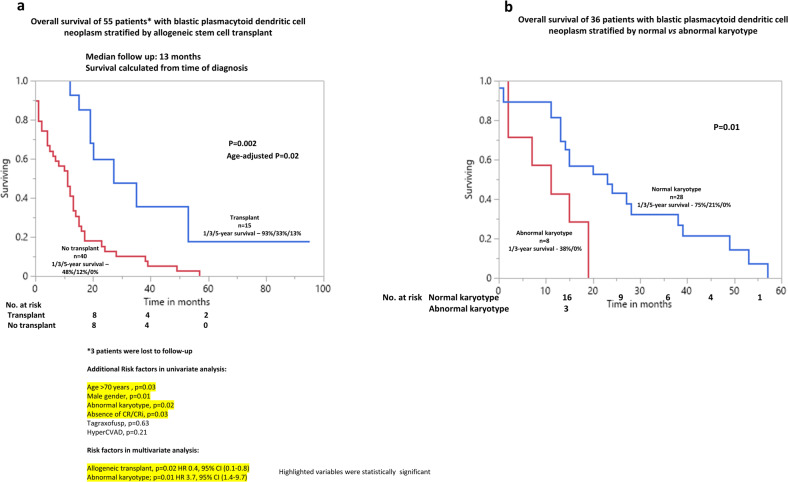
Overall survival of Mayo Clinic patients with blastic plasmacytoid dendritic cell neoplasm. **a** Overall survival of 55 patients with blastic plasmacytoid dendritic cell neoplasm stratified by allogeneic stem cell transplant. **b** Overall survival of 36 patients with blastic plasmacytoid dendritic cell neoplasm stratified by normal vs abnormal karyotype.

The current study suggests prognostic relevance for abnormal karyotype in patients with BPDCN. The reported incidence of abnormal karyotype in BPDCN is highly variable (7 to 66%) [2, 6, 8, 10, 14], moreover cytogenetic studies were performed in only a subset of patients using conventional karyotyping, with or without fluorescence in situ hybridization (FISH). In a prior report on cytogenetics in BPDCN, recurrent abnormalities including 5q, 12p, 13q, 6q, 15q and monosomy 9 were documented in 14 of 21 (66%) patients, while in another study, 14 of 21 (67%) patients displayed biallelic loss of 9p21.3 (*CDKN2A/CDKN2B*) on array-based comparative genomic hybridization [14, 15]. Although most contemporary studies, including a large international survey of 398 BPDCN patients with abnormal karyotype in 54%, have been unable to show a consistent relationship between abnormal karyotype and survival [8], a trend towards inferior survival was noted in one study [2]. In the particular multi-center study (*n* = 59), information on karyotype was available in 37 patients of which 13 (35%) had abnormal karyotype including -Y, and 2-year survival rates were 25% vs 61% for abnormal vs normal karyotype, respectively, (*p* = 0.15) [2]. In terms of the prognostic impact of specific abnormalities, only biallelic loss of 9p21.3 has been shown to be associated with inferior survival in a small series of 21 patients with BPDCN [15].

The current study also confirms a short-term survival advantage from ASCT in patients with BPDCN as in a Moffitt Cancer Center study (*n* = 49) [10], and an international multi-center survey (*n* = 398) [8]. In the latter study, patients that received lymphoma or acute leukemia treatments followed by ASCT demonstrated the best survival outcomes [8]. However, in our series, long-term survival with ASCT continued to be compromised with 3/5-year survival rates of 33 and 13%, respectively. All patients were in CR/CRi at the time of ASCT with the majority (80%) in first complete remission, regardless one-third succumbed to disease relapse. This is in contrast to observations from an MD Anderson study on ASCT in 17 patients with BPDCN (median age; 39 years), with 5-year survival rates of 40 and 80% for all patients and those in first complete remission, respectively [9]. These discrepancies might be a reflection of differences in age distribution of transplanted patients in the two studies. Our observations which require confirmation from collaborative studies, suggest an independent prognostic impact of karyotype and ASCT in patients with BPDCN.

## Supplementary information

supplemental material

checklist

## Data Availability

Data available by email gangat.naseema@mayo.edu
