# Supplementary material for: Abnormal karyotype is an independent predictor of inferior survival in Blastic Plasmacytoid Dendritic Cell Neoplasm (BPDCN)
Source: Blood Cancer J. 2023 Mar 13;13(1):35. doi: 10.1038/s41408-023-00812-y (PMC10008821; doi:10.1038/s41408-023-00812-y)
Supplement: Supplementary file 1 — supplemental material [file 41408_2023_812_MOESM1_ESM.docx]

Supplemental Table 1. Clinical characteristics at presentation, treatment details and outcomes for 8 patients with blastic plasmacytoid dendritic cell neoplasm and abnormal karyotype

| **Patient**  **No.** | **Age/Sex** | **Site of**  **disease** | **ELN 2017 Cytogenetic Risk**  **Karyotype*** | **Mutations** | **Initial therapy**  **Response** | **Relapse/**  **Therapy for relapse** | **Allogeneic**  **transplant** | **Overall survival**  **Dead or Alive** |
| --- | --- | --- | --- | --- | --- | --- | --- | --- |
| #1 | 62/F | Skin  LN  BM | Adverse  75-79,XXX,-X,-1,+3,add(3)(p21),-4,-4,add(4)(q31.1)x2,-5,-5,a  dd(6)(q13)x2,-7,-7,-8,-8,-9,-9,-10,-11,-11,-12,der(12)add(12  )(p11.2)del(12)(q13q15),-13,-13,-14,-15,der(15)t(15;16)(p11.  2;q11.1),-16,add(16)(q22),-17,add(17)(q21),add(17)(q23),-18,  -19,-22,-22,+7-12mar[cp7]/46,XX[13] | N.A | Tagraxofusp  CR | Post-transplant  Clinical trial | Mismatched unrelated donor  transplant | 19 months  Dead |
| #2 | 20/M | Skin  BM  CNS | Intermediate  46,XY del(12)(p11.2) [20] |  | Hyper CVAD  CR | no | Matched unrelated donor  transplant | 19 months  Dead |
| #3 | 65/M | BM | Intermediate  47,XY,+13[9]/46,XY[11] | *RUNX1*  *SRSF2* | Azacitidine+  venetoclax  CR | Tagraxofusp | no | 2 months  Dead |
| #4 | 76/M | BM  CNS | Adverse  46,XY,der(7;13)(q10;q10),del(12)(p11.2),-13,+16[10]/46,XY[10] | N.A | 7 (cytarabine)  +  3 (daunorubicin)  CRi | Hyper-CVAD | no | 7 months  Dead |
| #5 | 48/M | Skin  LN  BM | Adverse  45, XY, del(6)(q23 q25), der(7) t(7:12)(p13:q13), -12, del(18)(q21), -20, i(21)(q10), +mar [6] | N.A. | Mitoxantrone  +  Cytarabine  CR | N.A. | no | 15 months  Dead |
| #6 | 73/M | Skin  BM | Intermediate  45,XY,der(12;13)(q10;q10)[3]/  46,XY[17] | N.A. | Radiation  CR | Yes  None | no | 11 months  Dead |
| #7 | 67/M | Skin  LN  BM | Intermediate  47,XY,+8[3]/46,XY[17 | N.A. | None | - | no | 1 month  Alive |
| #8 | 70/M | Skin  LN  BM  CNS | Adverse  41-47,XYY?c,-6,-9[2],+mar[cp3] | N.A. | Cyclophosphamide  Etoposide  Bleomycin  PR | - | no | 2 months  Dead |

*Karyotype was performed on bone marrow

Abbreviations: ELN-European Leukemia Net, LN- lymph node, BM- bone marrow, N.A.- not available. CR- complete remission, CNS- central nervous system, Hyper CVAD- hyper fractioned cyclophosphamide, vincristine, doxorubicin, dexamethasone, alternating with high-dose methotrexate and cytarabine, CRi – complete remission with incomplete count recovery, PR- partial remission.

Supplemental Table 2. Details of cytogenetic abnormalities at the time of relapse for 5 patients

with blastic plasmacytoid dendritic cell neoplasm

| **Patient**  **No.** | **Age/Sex** | **Site of**  **disease** | **Cytogenetics**  **At diagnosis** | **Cytogenetics**  **At relapse** |
| --- | --- | --- | --- | --- |
| #1 | 69/M | Skin  BM | 46,XY[20] | 46,XY,der(4;12)(p10;p10),add(16)(q12),+mar[5]/46,XY[15] |
| #2 | 72/M | Skin  LN  BM  CNS | 46,XY[20] | 46,XY,add(19)(p13.3)[17]/46,XY[3] |
| #3 | 71/M | Skin  LN  BM  CNS | 46,XY[20] | 46,XY,add(4)(q12),add(6)(q21),add(8)(q24.1),del(12)(p11.2p13),add(16)(p13.1)[17]/46,XY[3] |
| #4 | 67/M | Skin  LN  BM | 46,XY[20] | 45,XY,der(3)t(1;3)(p22;p21),-6,der(8)t(8;13)(p12;q14),der(12)t(6;12)(p21;p13),del(17)(p11.2)[7]/46,XY[6] |
| #5 | 77/M | Skin  LN  BM | 45,X,-Y[12]/46,XY[8] | 44,XY,add(1)(q21),der(4)t(1;4)(q21;q21),-9,-13[9]/46,XY[11] |
